# Understanding solute effect on grain boundary strength based on atomic size and electronic interaction

**DOI:** 10.1038/s41598-020-74065-1

**Published:** 2020-10-08

**Authors:** Zhifeng Huang, Ping Wang, Fei Chen, Qiang Shen, Lianmeng Zhang

**Affiliations:** State Key Laboratory of Advanced Technology for Materials Synthesis and Processing, Wuhan University of Technology, Wuhan, 430070 People’s Republic of China

**Keywords:** Structural materials, Theory and computation

## Abstract

Solute segregating to grain boundary can stabilize the microstructure of nanocrystalline materials, but a lot of solutes also cause embrittlement effect on interfacial strength. Therefore, uncovering the solute effect on grain boundary strength is very important for nanocrystalline alloys design. In this work, we have systematically studied the effects of various solutes on the strength of a Σ5 (310) grain boundary in Cu by first-principle calculations. The solute effects are closely related to the atomic radius of solutes and electronic interactions between solutes and Cu. The solute with a larger atomic radius is easier to segregate the grain boundary but causes more significant grain boundary embrittlement. The weak electronic interactions between the *s*- and *p*-block solutes and Cu play a very limited role in enhancing grain boundary strength. While the strong *d*-states electronic interactions between transition metallic solutes and Cu can counteract embrittlement caused by size mismatch and significantly improve the grain boundary strength. This work deepens our understanding of solute effects on grain boundary strength based on atomic size and electronic interactions.

## Introduction

Solute segregation plays a very important role in governing the chemical composition and local structure of grain boundary, resulting in altering the mechanical, functional, and kinetic properties of nanocrystalline materials^1–3^. A number of previous works have shown that the thermal stability of nanocrystalline materials can be significantly improved by grain boundary segregation^4–8^. For example, Liu et al. showed that the segregation of metallic solutes can reduce the grain boundary energy to prevent nano grain growth^5^. Millett et al. found that the grain boundary stability can be improved by increasing solute coverage and atomic radius mismatch^6^. Although solute segregation can improve the thermal stability of grain boundary, it does not always play a good role in interfacial strength^9–11^. For example, experimental works have shown that Zr^12,13^, Nb^14^, Mo^15^, and Ta^16^ can enhance the mechanical strength but Bi^17,18^ causes brittle fracture of nanocrystalline Cu.

One important objective of grain boundary segregation engineering is to find suitable solutes which can improve the thermal stability of materials while avoiding embrittling effects^1,2,19^. Understanding the solute effect on grain boundary stability and strength mechanism is very important for guiding experimental alloy design. Murdoch et al. presented a Miedema model for estimation of grain boundary segregation enthalpy for thousands of binary alloys, which provides important guidance for processing thermally stable nanocrystalline alloys^1^. Wu et al. reported that the strengthening effect of transition metallic solutes on W grain boundaries depends on the atomic radius of solutes^20^. Schweinfest et al. concluded that the embrittlement effect of Bi on the grain boundary in Cu is caused by the big misfit in the atomic radii between Cu and Bi^18^. However, Duscher et al.^17^ and Kang et al.^21^ held the opposite view that Bi embrittles Cu grain boundaries because of electronic interactions. Although a lot of works have studied the key factors affecting grain boundary strength, the mechanisms behind solute effects are still controversial.

Herein, in this work, we focus on uncovering the mechanism behind the solute segregation and strengthening/weakening effects on a common Σ5 (310) grain boundary in Cu^22^ using first-principles calculations. To give full consideration of different effects caused by various atomic radius and valence electronic configuration of solutes, we have considered 48 common metallic solutes from different blocks in the periodic table of elements. We first calculate the segregation energies of various solutes at different substitutional sites at the grain boundary. We then calculate the strengthening energies of grain boundary models with the lowest segregation energies, to determine the strengthening or weakening effects of solutes on grain boundary. The results indicate that both the atomic radius of solutes and electronic interactions between solutes and Cu have important roles in altering the grain boundary strength.

## Computational methods and details

**Figure 1:**
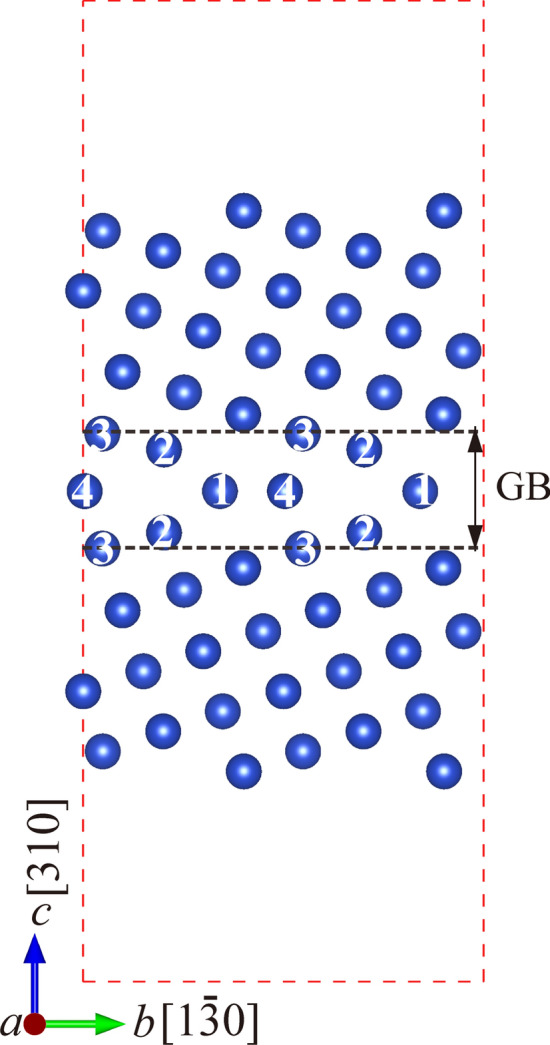
Schematic illustrations of the Σ5 (310) grain boundary in Cu and the substitutional sites at the interface.

Figure 1 presents the Σ5 (310) grain boundary model in face-centered cubic Cu, where the numbers 1, 2, 3, and 4 denote for the four different substitutional sites at the interface^23^. The dimensions of the grain boundary model are 7.267 Å × 11.490 Å × 28.086 Å, comprising 27 atomic layers and a vacuum layer of 12 Å. All calculations were performed based on density functional theory implemented in the Vienna *ab*-*initio* simulation package (VASP)^24^ using the projector augmented wave approach and the Perdew–Burke–Ernzerhof exchange–correlation generalized gradient approximation functional^25,26^. The plane-wave cutoff energy of 350 eV, *k*-point meshes of 3 × 2 × 1, energy convergence of 10^–5^ eV/atom, and force convergence of 0.01 eV/Å were employed in the calculations^23^. To give a comprehensive understanding of solute effects on segregation ability and grain boundary strength, we considered 48 common metallic solutes with different atomic radius and valence electronic configurations.

The segregation ability of a solute X can be characterized by segregation energy (*E*_seg_)^23,27,28^:

1 $${E}_{seg}=\left({E}_{GB+X}-{E}_{GB}\right)-\left({E}_{bulk+X}-{E}_{bulk}\right)$$

where *E*_GB_ and *E*_GB+X_ are the total energies of clean and doped grain boundaries, and *E*_bulk_ and *E*_bulk+X_ are the total energies clean and doped bulk Cu, respectively. The bulk model has the same dimension and number of atoms as the grain boundary model^23^. Negative segregation energy indicates the solute segregating into the site is energetically favorable^28^.

The effect of the solute X on the grain boundary strength can be predicted by the strengthening energy (*E*_str_)^9,20^:

2 $${E}_{str}=\left({E}_{GB+X}-{E}_{GB}\right)-({E}_{FS+X}-{E}_{FS})$$

where *E*_FS_ and *E*_FS+X_ are the total energies of the clean and doped (310) Cu free surfaces. The free surface model was generated by removing the upper crystal in the grain boundary model^23^. The site of solute X at the free surface is the same as the site at the grain boundary. A negative value of strengthening energy means a strengthening effect on grain boundary strength while a positive value suggests a weakening effect. Furthermore, to understand the grain boundary strengthening/weakening resulting from the local structural deformation and the electronic interactions change after introducing a solute into the boundary, we divided the strengthening energy into mechanical and chemical contributions^29–31^.

## Results and discussion

### Segregation energy

**Figure 2:**
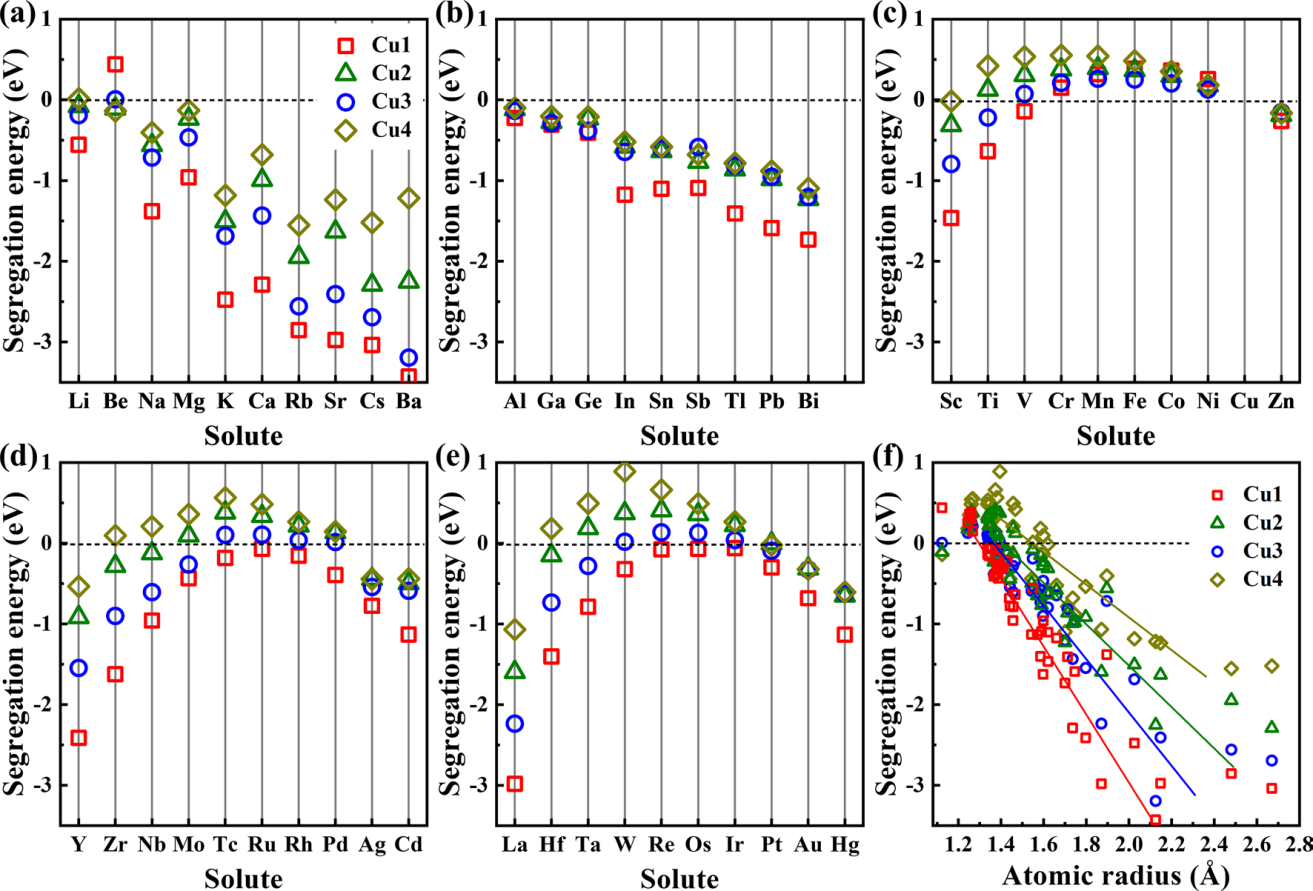
(**a**–**e**) The segregation energies of solutes occupying different substitutional sites at the grain boundary. (**f**) The relationship between the segregation energy and the atomic radius of solutes.

Figure 2 presents the segregation energies of solutes occupying different substitutional sites at the grain boundary. We can see that all the segregation energies of Cr, Mn, Fe, Co, and Ni at the four substitutional sites are positive, indicating that these solutes will not segregate to the grain boundary and tend to stay in grains. For solutes with negative segregation energies, except Be at Cu4 site is the lowest, the segregation energies are lowest when the solute at Cu1 site. Besides, the segregation energy data from Fig. 2c–e show a similar change trend from the left to the right, suggesting that the segregation ability of the transition metals exhibit similar periodic properties. To uncover the factor influencing the segregation ability, we plotted the relationship between the segregation energy and the atomic radius of solutes in Fig. 2f. We can see that the values of segregation energies are positive or close to zero when the atomic radii of solutes are smaller or close to the atomic radius of Cu (1.17 Å^32^). The segregation energy decreases as the atomic radius increases when the solute is much larger than Cu. The results indicate that solutes with a smaller or similar atomic size of Cu have no or very limited segregation ability to the grain boundary, while solutes with a larger size than Cu prefer to segregate to the interface. Besides, the relationship between the segregation energy and the site volume at the grain boundary is also plotted in Fig. 2f, as guided by the lines. The site volume reflects the space size of a solute atom can be accommodated. We can see that the negative slope of solute occupied Cu1 site is the most, followed by Cu3, Cu2, and Cu4, which is in the same order as the site volumes of Cu1 (15.23 Å^3^), Cu3 (13.14 Å^3^), Cu2 (12.91 Å^3^) and Cu4 (12.45 Å^3^). Therefore, both the large atomic radius of solutes and the large site volume of Cu at the grain boundary contribute to the segregation of solutes.

### Strengthening energy

**Figure 3:**
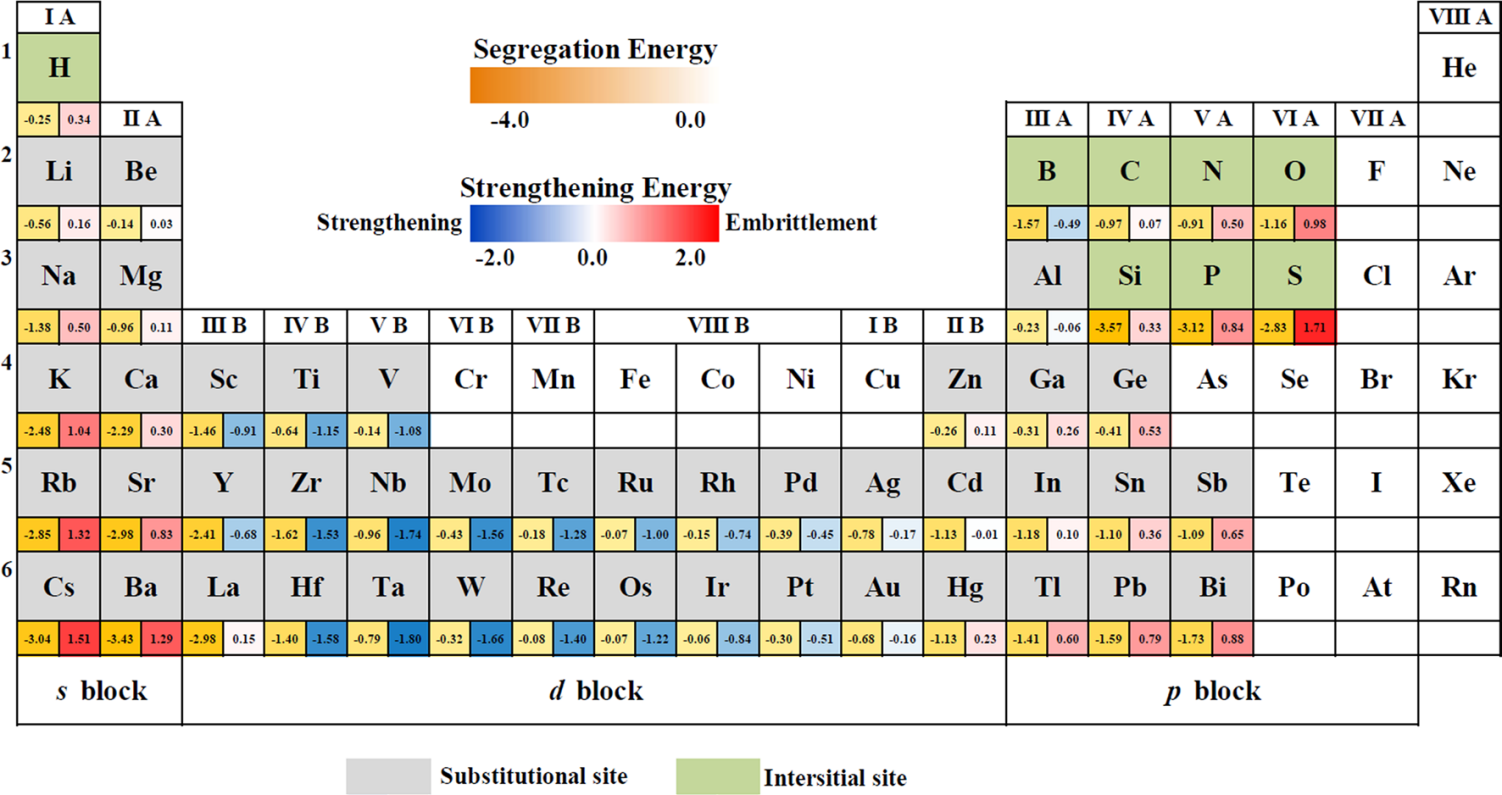
The strengthening and segregation energies of grain boundaries with solute at the preferred site at the grain boundary.

Figure 3 presents the strengthening energies calculated by the grain boundary models with solute at the preferred site, as well as the lowest segregation energies of solutes. The energies data of nonmetallic impurities from the previous work^23^ are also shown here for the comprehensive understanding of the effects of various solutes. The red color (positive value) denotes the dopant will weaken the grain boundary strength, but the blue color (negative value) indicates the dopant will enhance the grain boundary strength. We can find that the strengthening energies of solutes coming from the *s* and *p* blocks of the periodic table of elements are positive except for B, while the strengthening energies of transition metallic solutes are negative, except Zn, La and Hg are small positives. The results indicate that solutes coming from the *s* and *p* blocks will weaken the grain boundary except for B, but the *d*-block solutes will enhance the grain boundary strength except for Zn, La, and Hg.

To improve the thermal stability of materials but avoiding embrittlement is very important for the research of grain boundary segregation engineering^2,19^. Figure 3 gives an overview of the segregation ability and strengthening/weakening effect of solutes on the Cu grain boundary. We can see that the segregation energies of metallic solutes from the *s* and *p* blocks are generally lower than that of solutes from the *d* block, which is due to the relatively larger atomic radius of *s*- and *p*-block solutes than *d*-block solutes. Previous works have shown that grain boundary energy decreases with increasing the atomic radius of solutes, as a result, improves the thermal stability of nanocrystalline materials^4–6^. Herein, solutes from the *s* and *p* blocks can be good options to prepare Cu-based binary nanocrystalline alloys with great thermal stability. However, when considering the effect of solutes on the grain boundary strength, more attention needs to be paid to the *d*-block solutes. We can see from Fig. 3 that the segregation and strengthening energies are relative lower of the *d*-block solutes at the front of each period (such as Ti, V, Zr, Nb, Mo, Tc, Hf, Ta, W, Re, and Os) comparing to the later solutes at the same period, indicating these solutes can improve both the stability and strength of grain boundary. Our simulation results are in good agreement with published experiment works. For example, nanocrystalline alloys such as Cu–Zr^12,13^, Cu–Nb^14^, Cu–Mo^15^, and Cu–Ta^16^ have more stable microstructures and better mechanical properties than nanocrystalline Cu. Nanocrystalline Cu-Bi alloys exhibit good thermal stability however Bi often leads to grain boundary embrittlement, resulting in cracks in materials^17,18^. Therefore, Fig. 3 can provide a theoretical base for solute selection for the design of nanocrystalline Cu-based alloys.

### Mechanical and chemical contributions to grain boundary strength

**Figure 4:**
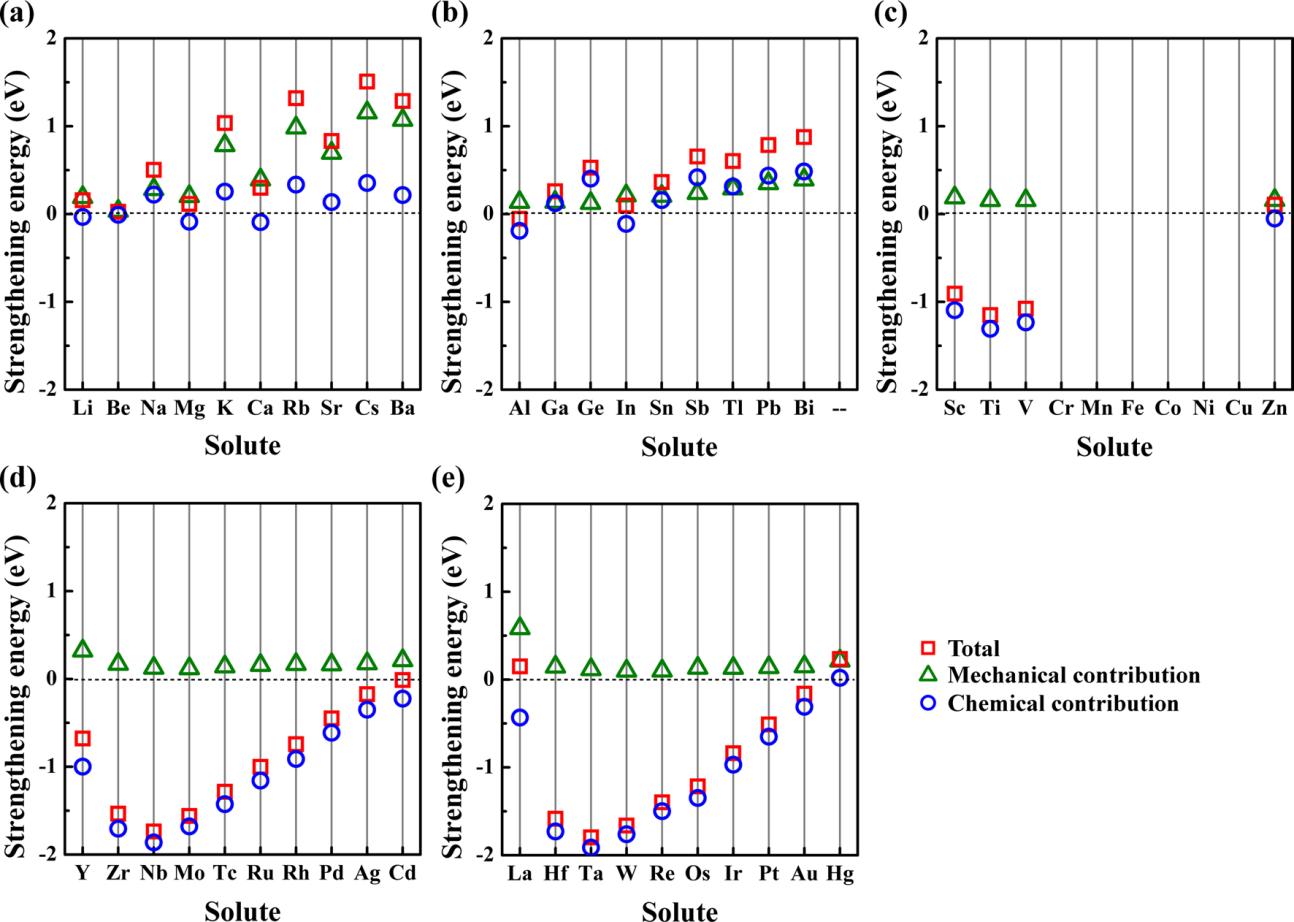
The mechanical and chemical contributions of strengthening energy of grain boundaries with solute at the preference site.

With the overall effect of solutes on the grain boundary strength shown in Sect. 3.2, we now turn our attention to the mechanical and chemical contributing mechanism. Figure 4 shows the mechanical and chemical contributions to the strengthening energy. We can see that the mechanical contributions are positive for all solutes, indicating mechanical contribution always plays the weakening effect on grain boundary strength. However, the chemical contributions of solutes are various a lot with different solutes. For example, the chemical contributions of dopants coming from the *s* and *p* blocks are slightly positive or close to zero, indicating that the electronic interaction between these solutes and Cu has a very limited effect on grain boundary strength. The chemical contributions of the *d*-block solutes are negative, indicating that the chemical interaction between transition metallic solutes and Cu can improve the grain boundary strength. Besides, we can see the similar change trend of the strengthening energy as well as the mechanical and chemical contributions of the transition metallic solutes from different periods.

**Figure 5:**
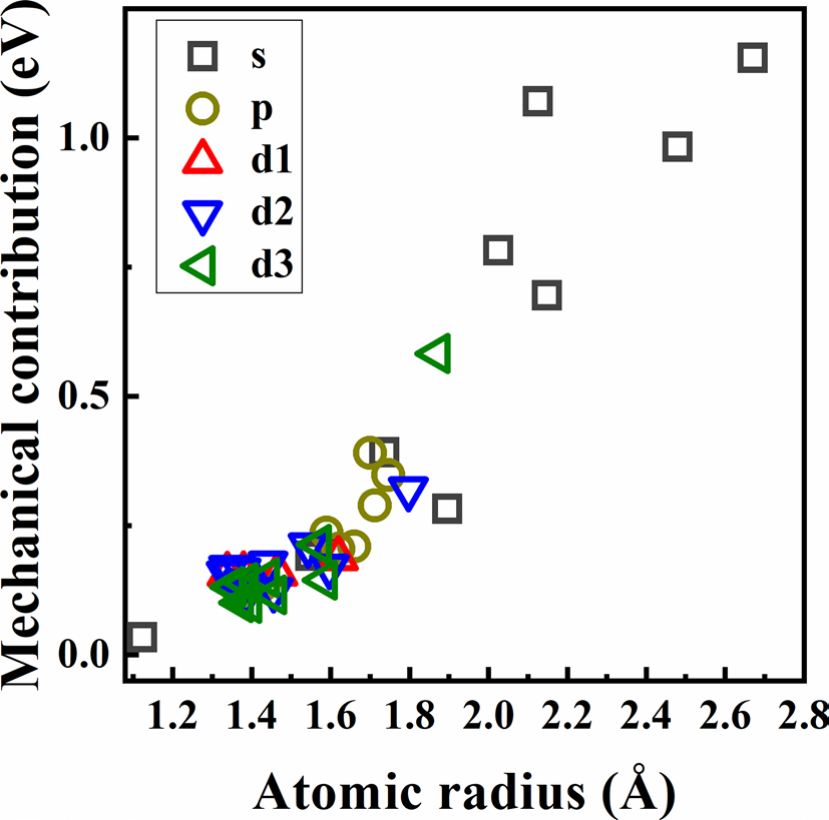
The relationship between the mechanical contribution and atomic radius of solutes.

To clarify the mechanism of mechanical and chemical contributions, we turn to study the effect coming from the atomic size of solutes and electronic interaction between solutes and Cu. Figure 5 shows the relationship between the mechanical contribution and the atomic radius of solutes. We can see that the mechanical contribution increases with increasing the atomic radius of solutes, indicating that a larger solute will cause a more embrittling effect on grain boundary strength. The mechanical contribution is mainly due to the deformation of grain boundary structure caused by replacing the Cu atom with the solute atom^29^. Therefore, a large solute causing the large structural deformation at the interface will lead to significant embrittlement to the grain boundary.

**Figure 6:**
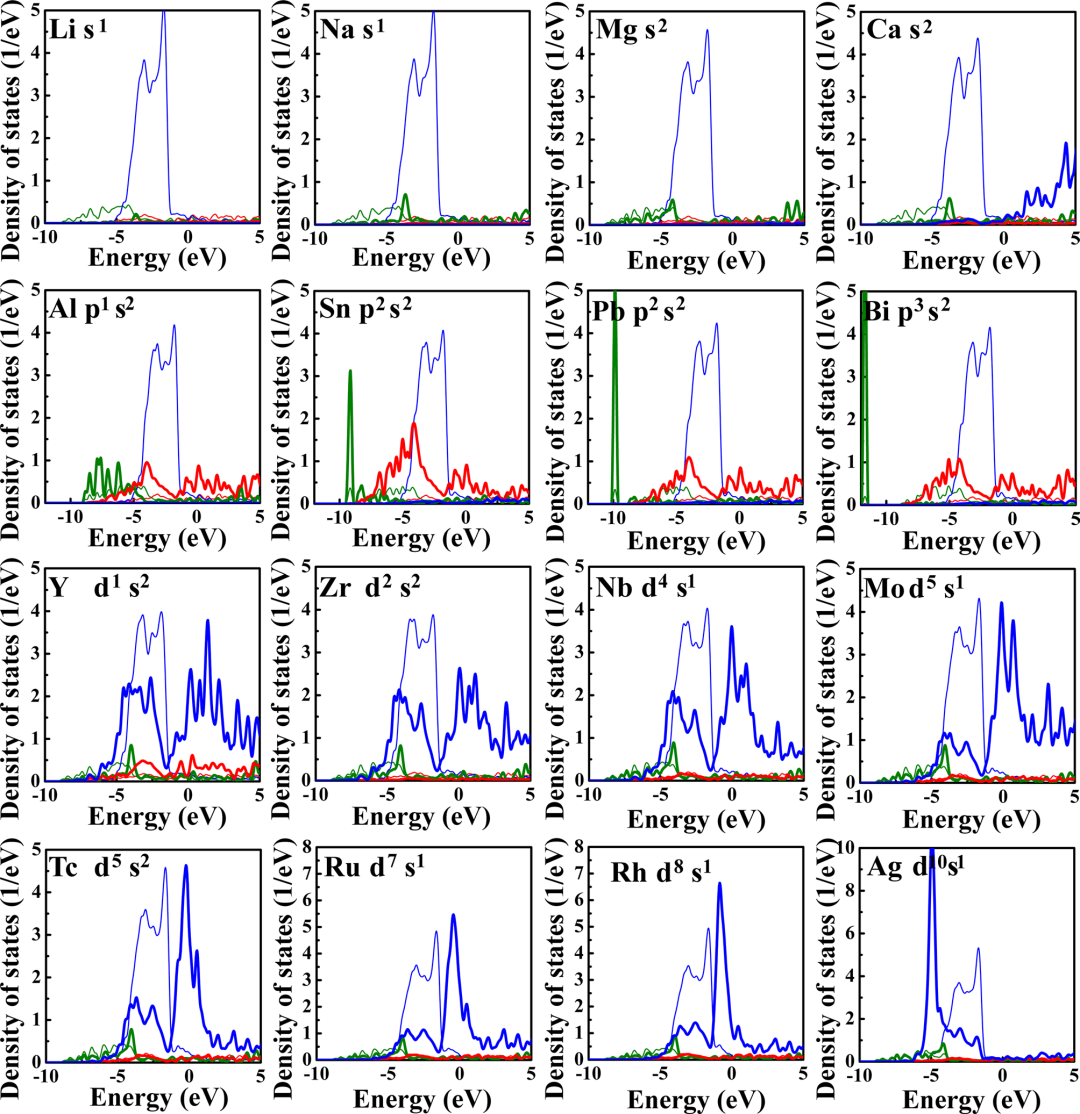
The density of states data of the solute and the nearest Cu at the interface. The data of the solute and the Cu are shown as thick curves and thin curves, respectively.

Considering the similarity of the valence electronic configurations, we chose Li, Na, Mg, and Ca in *s* block, Al, Sn, Pb, and Bi from *p* block, and the fifth periodic transition metallic solutes to investigate the electronic interactions of these solutes and Cu. Figure 6 presents the density of states data of solutes and the nearest Cu atoms at the grain boundary. For the *s*-block solutes, the peak in the interval of − 5–0 eV is composed of *d* states electrons of Cu and little content of *s* states electrons of solutes, suggesting that there is almost no electronic interaction between the *s*-block solutes and Cu. The little electronic interaction has a very limited effect on the grain boundary strength, which explains why the chemical contribution of the *s*-block solutes is close to zero as shown in Fig. 4a. For the *p*-block solutes, there is a partial overlap between the *p* states electrons of solutes and *d* states electrons of Cu. But the overlap area is relatively small compared with the main peak, indicating the weak electronic interaction between the *p*-block solutes and Cu. A similar electronic interaction between the *p*-block solutes and Cu have also been found in previous works when investigating the electronic interactions between some nonmetallic impurities and Cu^23,33^. Such electronic interactions electron interactions exhibit the polar bonding effects, which have been shown to weaken the grain boundary strength^23,33^. That why the values of chemical contributions of *p*-block solutes in Fig. 4b are positive. For the *d*-block solutes, we can see that the main peak is mainly composed of the *d* states electrons of solutes and the *d* states electrons of Cu, while the contribution of *s* and *p* state electrons is very limited, suggesting that the strengthening effect of *d*-block solutes on the grain boundary is dominated by the *d* states electronic interaction between solutes and Cu. For example, the overlap degrees between the peak of the *d* states of Cu and the peak of the *d* states of solutes at the front of the fifth period (such as Y, Zr, Nb, Mo, and Tc) are significantly higher than that of solutes at the back of the same period (such as Ru, Rh, Ag). The *d* orbital of the solutes at the front of the same period has the lower occupation degree of which the electrons are easier to interact with the *d*-orbital electrons of Cu. For example, the peak shapes of the *d*-orbital density of states of these solutes in the range of − 5–0 eV are similar to that of Cu, indicating that the electronic interactions are strong between there solutes and Cu. However, when increasing the number of electrons in the *d* orbital of solutes, the mutual constraint between the spin electrons increases, resulting in decreasing electronic interaction between solutes and Cu. For example, the peak overlaps between the *d* orbital density of states of solutes at the back of the same period (such as Ru, Rh, Ag) and Cu are lower, and the distribution of *d*-orbital electrons becomes more and more localized. Therefore, as shown in Fig. 4c–e, the chemical contributions of solutes at the front of each period have a strengthening effect on the grain boundary strength, while the strengthening effect becomes weaker and weaker when increasing the atomic number in the same period.

Both the atomic radius and electronic configuration of solutes play important effects on grain boundary strength. Due to the large atomic radius of *s*-block solutes and the limited electronic interaction between *s*-block solutes and Cu, the *s*-block solutes will weaken the grain boundary strength. The large atomic radius of *p*-block solutes and the polar electronic interaction between them and Cu will cause an embrittlement effect on grain boundary, which explains the experimental observations that the *p*-block solute Bi leads to grain boundary embrittlement in nanocrystalline Cu–Bi alloys^17,18^. The atomic radius misfit between *d*-block solutes and Cu will also cause embrittlement. However, such an effect could be offset when the strong electronic interaction occurring between the *d*-block solutes and Cu. These results deepen our understanding of the experimental observations that nanocrystalline Cu–Zr^12,13^, Cu–Nb^14^, Cu–Mo^15^ and Cu–Ta^16^ alloys show better mechanical strength than nanocrystalline Cu from the electronic scale.

## Conclusions

This work systematically studied the segregation ability and strengthening/embrittlement effect of solutes with different atomic radius and valence electronic configurations on a Cu Σ5 (310) grain boundary by first-principles simulations. The solute with a larger atomic radius has a stronger segregation ability to occupy the Cu site with a larger volume. The strengthening/embrittlement effect is closely related to the atomic radius of solute and electronic interaction between the solute and Cu. The solute with a large atomic radius will cause large deformation at the grain boundary resulting in weakening the interfacial strength. The strong *d*-states electronic interaction between the transition metallic solute and Cu can significantly improve the grain boundary strength. However, the weak electronic interactions between the *s*- and *p*-block solutes and Cu play a limited role in enhancing grain boundary strength. This work deepens the understanding of segregation and strengthening/embrittlement effects of solutes and the systematic data can guide the design of Cu-based alloys.
